# An Odorant Receptor from the Proboscis of the Cotton Bollworm *Helicoverpa armigera* (Lepidoptera: Noctuidae) Narrowly Tuned to Indole

**DOI:** 10.3390/insects13040385

**Published:** 2022-04-13

**Authors:** Mengbo Guo, Xueting Ren, Yang Liu, Guirong Wang

**Affiliations:** 1 Shenzhen Branch, Guangdong Laboratory for Lingnan Modern Agriculture, Genome Analysis Laboratory of the Ministry of Agriculture and Rural Affairs, Agricultural Genomics Institute at Shenzhen, Chinese Academy of Agricultural Sciences, Shenzhen 518120, China; guomengbo@caas.cn; 2 State Key Laboratory for Biology of Plant Diseases and Insect Pests, Institute of Plant Protection, Chinese Academy of Agricultural Sciences, Beijing 100193, China; renren0712@163.com (X.R.); yangliu@ippcaas.cn (Y.L.)

**Keywords:** odorant receptor, proboscis, *Helicoverpa armigera*, functional identification, indole

## Abstract

**Simple Summary:**

Odorant receptors (ORs) are at the core of the high-efficiency and sensitive olfactory system in insects. The expression and specific function of ORs largely contribute to the habits and speciation of one species. Although being predominantly expressed in the antennae, ORs in non-olfactory organs are suggested to have particular roles in promoting the reproduction or host fitness of insects. Our previous work has identified four ORs in the mouthpart organs of *Helicoverpa armigera*. Here, we amplified the full-length sequences of HarmORs from the proboscis. Further functional characterization suggested that HarmOR30 narrowly tuned to indole, the vital nitrogen-containing compounds that mediate tritrophic interactions. Our study deepens the insight into the olfactory perception of *H. armigera*, and explored a candidate functional receptor target for studying the interaction between insects and their plant hosts.

**Abstract:**

*Helicoverpa armigera* is a serious agricultural pest with polyphagous diets, widespread distribution, and causing severe damage. Among sixty-five candidate ORs in *H. armigera*, the co-receptor HarmOrco and three specific ORs with partial sequences were identified to be expressed in the proboscis by our previous work, whereas their exact function is not known yet. In this study, we first confirmed the expression of these ORs in the proboscis by full-length cloning, which obtained the complete coding region of HarmOrco, OR24, and OR30. We then performed functional identification of HarmOR24 and OR30 by co-expressing them respectively with HarmOrco in *Xenopus* oocytes eukaryotic expression system combined with two-electrode voltage-clamp physiology. By testing the response of HarmOR24/OR30-expressing oocytes against eighty structural-divergent compounds, respectively, HarmOR30 was characterized to narrowly tune to indole and showed a specific tuning spectrum compared to its ortholog in *Spodoptera littoralis*. As indole is a distinctive herbivore-induced plant volatile and floral scent component, HarmOR30 might play roles in foraging and mediating the interactions between *H. armigera* with its surrounding environment.

## 1. Introduction

As the dominant group of invertebrates in most terrestrial ecosystems of the world, insects perform vital ecological functions through their feeding activities in either mutualistic or antagonistic ways like pollination, decomposition, phytophagy, predation, and parasitism [1]. For accommodating various feeding niches, insects from different taxa have evolved diverse forms of feeding organs on the mouthparts [2,3]. In glossatan Lepidoptera, almost all the adults of moths and butterflies have a siphonate proboscis, an elongated feeding tube attached to the head, and is tightly coiled up and stored under the heads during rest position or stretched while feeding [4,5]. In addition to the function in feeding, the proboscis is reported to have sensory functions such as chemoreception and mechanoreception [4]. The external surface of the proboscis is equipped with various types of sensilla associated with chemo- and/or mechanosensilla, which are thought to process gustatory or mechanical information during food intake [6]. It is worth pointing out that the proboscis, and other non-olfactory organs, also have been reported to possess olfactory sensing function, despite antennae being the primary olfactory organ for odorant detecting. In the Diptera *Anopheles gambiae*, the labellum of the proboscis was indicated to detect a small spectrum of human-related odorants [7]. In the Lepidoptera hawkmoth *Manduca sexta*, olfactory neurons were identified in the proboscis sensilla for sensing the floral scent benzyl acetone (BA) of wild tobacco [8].

In insects, the transmission of initial odorant signals is mainly mediated by ORs, which are expressed on the dendrite membrane of olfactory sensing neurons in the sensilla [9]. Insect ORs share similar seven-transmembrane domains with G protein-coupled receptors in vertebrates and nematodes but have an inverted topology with an intracellular N-terminus and an extracellular C-terminus [10]. Two sub-families of ORs have been identified in insects: (1) the ligand-specific ORs, which own lots of members in one species and their sequences are divergent within or between organisms, and (2) the co-receptors Orco, whose sequences are highly conserved among divergent insect orders. The co-receptors are not known to respond to any naturally occurring ligands. Ligand-specific ORs play roles by co-expressing with Orco to form heteromeric complexes as ligand-gated ion channels [11,12]. Characterization of the OR family in numerous species has largely taken our insight into the chemoreception of insects. Previous studies have reported the expression of candidate ORs in the proboscis of several lepidopteran species. Studies in *Heliothis virescens* have revealed the presence of candidate ORs in the proboscis by using tissue expression surveys [13]. Likewise, by performing transcriptomic sequencing on different tissues, transcripts of some ORs are also identified in proboscis and other tissues in several lepidopteran species such as *Spodoptera littoralis* and *Mythimna separata*, etc. [14,15].

Our previous works have identified sixty-five candidate ORs by transcriptomic sequencing in *Helicoverpa armigera*, a severe agricultural pest with herbivorous larvae and pollinated adults [16,17,18]. We have characterized the sensilla types of proboscis, which is equipped with nine subtypes of sensilla, including a wall-multiporous one that implies its olfactory sensing function [18]. By transcriptomic assays of the adult mouthpart organs (proboscis and labial palps), we identified the candidate chemosensory genes, including four ORs (HarmOrco, OR24, OR30, and OR58). In addition, the transcripts of these OR genes have been detected in the legs and antennae of both sexes [18]. Functional identification of forty-four HarmORs has been performed by screening the ligands of each OR against multiple odorant compounds, whereas no ligand of HarmOR24 or OR30 was identified [19]. We raise the question of what the function of these ORs is? Here, we focused on the proboscis and performed full-length sequence cloning of HarmORs, including co-receptor HarmOrco and three specific ORs HarmOR24, OR30, and OR58. By screening the ligands of each OR through heterologous expression in the *Xenopus* oocytes system combined with two-voltage clamp electrophysiology, we characterized the function of HarmOR30 for sensing indole. As one of the most important nitrogen-containing aromatic compounds, indole has been reported to play crucial ecological roles in manipulating tritrophic interactions [20,21]. The indole-sensing function of HarmOR30, which is expressed in non-olfactory organs, might mediate the host-plant recognition of *H. armigera*.

## 2. Materials and Methods

### 2.1. Insects

The *H. armigera* moths used in this study come from a laboratory colony, which has been bred for more than ten generations under laboratory conditions (16:8 h light: dark photoperiod, 27 °C ± 1 °C and 60–65% RH) at the Institute of Plant Protection, Chinese Academy of Agricultural Sciences, Beijing, China. Male pupae were kept separately from females before eclosion. The moths were fed on 10% honey water until they were used for experiments.

### 2.2. RNA Extraction, cDNA Synthesis, and HarmORs Cloning

The tissues of adults were carefully dissected from the 1- to 3-day-old moths of both sexes and then stored in liquid nitrogen immediately. The total RNA of proboscis was extracted following the manufacturer’s instructions using TriZol reagent (Invitrogen, Carlsbad, CA, USA.). After extraction, the quality of RNA was assessed with a NanoDrop ND-2000 spectrophotometer (Thermo Fisher Scientifc, Waltham, MA, USA) and in a 1.2% agarose gel. The first-strand cDNA was synthesized from 1 μg of total RNA using an oligo-dT primer by TransScript^®^ One-Step gDNA (TransGen Biotech, Beijing, China) and following the manufacturer’s protocol. The product was either used directly for full-length cloning of HarmORs or stored at −70 °C.

For the full-length cloning of HarmORs, the coding region of each OR (HarmOrco, OR24, OR30, and OR58) was amplified by using PrimeSTAR HS DNA polymerase (Takara, Dalian, China) from the cDNA mixture (1:1) of the proboscis of both sexes by PCR using specific primers (Table 1) based on a reported sequence (Genbank: HarmOrco, MN399769; HarmOR24, MT479012; HarmOR30, MT479017; HarmOR58, XP_021199476.1). The PCR reaction was performed in 50 μL, including 25 μL of 2×PrimeSTAR mix, 1 μL of each primer (10 μM), 2 μL of cDNA template, and 21 μL of H_2_O. The reactions were carried out using a Veriti Thermal Cycler (Applied Biosystems, Carlsbad, CA, USA) under the following conditions: 98 °C for 1 min, 32 cycles of 98 °C for 10 s, 50 °C for 15 s, and 72 °C for 1.5 min, and 72 °C for 2 min. PCR amplification products were assessed with 1.2% agarose gel and cloned into the pEASY-Blunt vector (TransGen Biotech, Beijing, China).

### 2.3. Sequences Alignment of HarmORs and Transmembrane Domains Analysis

Sequences of the candidate ORs were aligned with MAFFT version 7 online program (2 February 2022, https://mafft.cbrc.jp/alignment/server/), using auto (FFT-NS-1, FFT-NS-2, FFT-NS-i, or L-INS-i; depends on data size) default parameters. The protein topologies and transmembrane domains of each HarmOR were predicted TOPCONS (2 February 2022, http://topcons.cbr.su.se/).

### 2.4. Heterologous Expression of HarmORs: cRNA Synthesis and Oocyte Microinjection

The coding region of each HarmOR24 and OR30 was subcloned into eukaryotic expression vector pT7TS by using ClonExpressII One Step Cloning Kit (Vazyme Biotech Co., Ltd., Nanjing, China). The specific primers bearing a Kozak consensus of each OR are listed in Table 1. cRNAs of HarmOR24, OR30, and the co-receptor HarmOrco were generated from linearized expression vectors by using the mMESSAGE mMACHINE T7 kit (Ambion, Austin, TX, USA) according to the manufacturer’s instructions. The expression vector of HarmOrco, which has been constructed in previous work [19], was used in this study. Then, the cRNA mixture of ORx/Orco (27.6 ng each) was injected (Nanoliter 2010, WPI Inc., Sarasota, FL) into single mature and healthy *Xenopus* oocytes (stage V–VII), which were dissected from female *Xenopus laevis* freshly and then treated with 2 mg/mL collagenase solution in 1 × Ringer’s buffer (2 mM KCl, 96 mM NaCl, 5 mM HEPES pH 7.6, and 5 mM MgCl2) at room temperature for 40 min to 1 h. After injection, oocytes were incubated with 1 × Ringer’s buffer, which was supplemented with 5% dialyzed horse serum, 50 mg/mL tetracycline, 100 mg/mL streptomycin, and 550 mg/mL sodium pyruvate, for 3–4 days at 18 °C.

### 2.5. Plant Volatile Compounds for Ligand Screening of HarmORs

Total eighty diverse volatile compounds (Supplementary Table S1), including short- and mid-chain carboxylic acids, amines, aldehydes, aromatics, terpenes, short-chain fatty acid derivatives, and nitrogen-containing heterocyclic compounds, were tested in this study. Stock solution (1 M) of each compound was prepared in dimethyl sulfoxide (DMSO) and stored at −20 °C. When performing ligands screening, each odorant was freshly prepared by diluting into the dosage of 10^−4^ M with 1 × Ringer’s buffer containing 0.8 mM CaCl_2_.

### 2.6. Whole-Cell Two-Electrode Voltage-Clamp Physiological Recording

The whole-cell current of a single oocyte to multiple plant odorants was recorded by OC-725C oocyte clamp (Warner Instruments, LLC, Holliston, MA, USA) at a holding potential of −80 mV. After the current return to baseline, each oocyte was exposed to each compound by rinsing with 1 × Ringer’s buffer containing 0.8 mM CaCl_2_. The data were acquired by Digidata 1440A and analyzed by pCLAMP 10.2 software (Axon Instruments Inc., Union City, CA, USA). Each compound was tested with 4–7 single cells to confirm the response profile. The tuning spectrum of OR was generated and analyzed by using GraphPad Prism 5 (GraphPad Software Inc., San Diego, CA, USA).

### 2.7. Phylogenetic Analysis of Lepidopteran ORs

The phylogenetic tree of 312 ORs from six lepidopteran species was constructed based on the maximum-likelihood method. Sequences alignment of the amino acid was performed using MAFFT (10 December 2021, https://www.ebi.ac.uk/Tools/msa/mafft/). The tree was constructed using RAxML version 8 with the Jones–Taylor–Thornton amino acid substitution model (JTT) and 1000 bootstrap replicates to assess node support.

## 3. Results

### 3.1. Full-Length Cloning of HarmOR Genes from the Proboscis

Our previous studies have identified four ORs in the proboscis, including the odorant co-receptor Orco with a complete open reading frame (ORF) and three partial sequences of HarmOR24, OR30, and OR58 [18]. To confirm the expression of these OR genes, we performed full-length cDNA cloning from proboscis based on reported sequences. The full-length of HarmOrco, OR24, and OR30, which encode a polypeptide of 400, 391, and 382 amino acid residues, respectively, were obtained successfully. We failed to clone the full-length sequence of HarmOR58, as no PCR product was obtained by using the specific primers. The possible reason might be the shallow expression quantity of this gene in the proboscis. The sequence of each OR gene is highly consistent with its reported sequence. The amino acid sequence of HarmOR24, OR30, and Orco shares 99.5%, 97.4%, and 100% identities with the reported reference sequence, respectively. The sequences of HarmOR24 and OR30 are shown in Supplementary Table S2. Transmembrane (TM) domains’ prediction of each OR indicated that they have a typical seven TM domains and extracellular C-terminus.

### 3.2. Functional Identification of HarmORs

**Figure 1:**
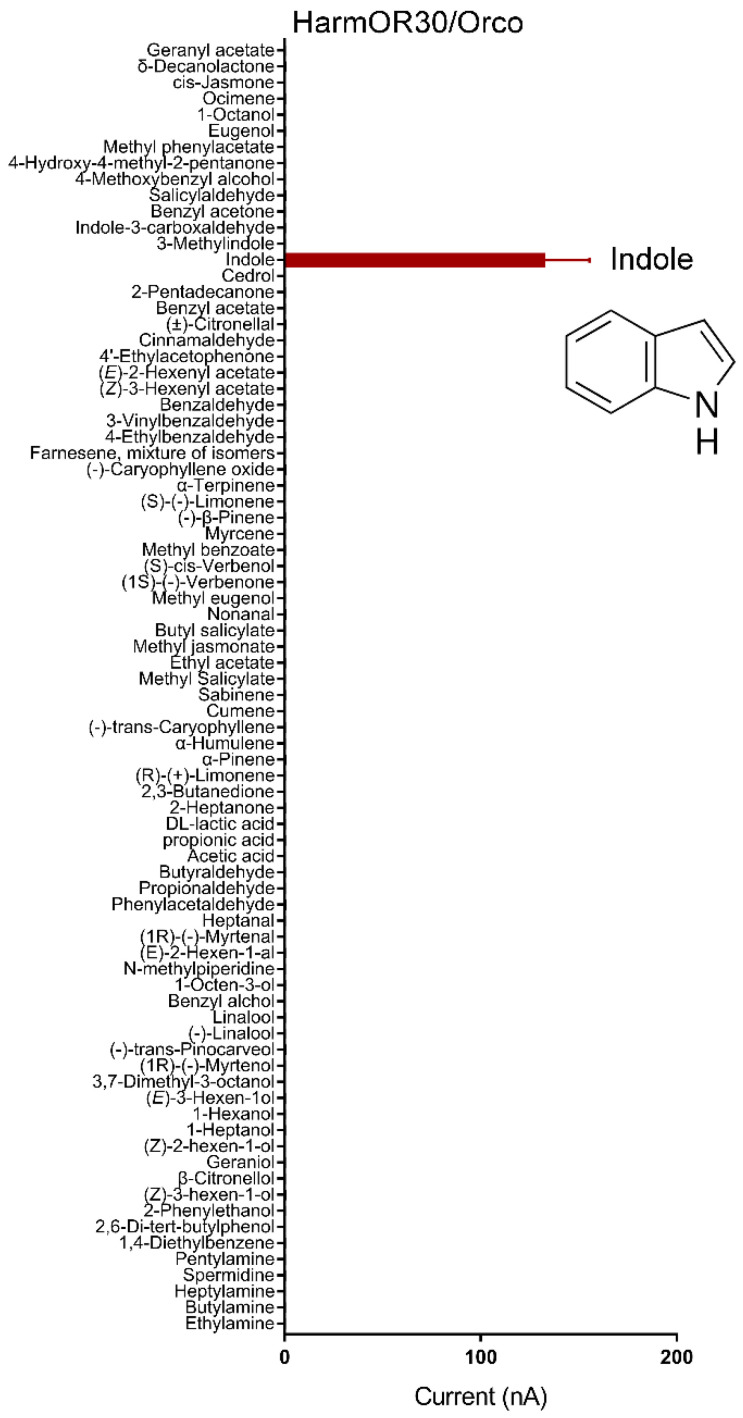
Functional characterization of HarmOR30/Orco in *Xenopus* oocytes against eighty compounds at the dosage of 10^−4^ M. The red column represents the response of HarmOR30 to indole.

In *H. armigera*, the function of forty-four HarmORs expressed in adult antennae has been screened against a panel of sixty-seven plant volatiles in our previous studies, where no ligand of OR24 and OR30 was characterized [19]. Considering the particular expressing pattern of these ORs, we suppose that they may respond to ecologically relevant compounds and then perform further functional identification in vitro. We first subcloned them into the eukaryotic expression vector to generate the cRNA of each OR, respectively. Then, the cRNA mixture of HarmORx/Orco was injected into *Xenopus* oocytes followed by a whole-cell two-electrode voltage-clamp physiological system. Each cell was tested against eighty plant volatile compounds from diverse categories, including short- and mid-chain carboxylic acids, amines, aldehydes, aromatics, terpenes, short-chain fatty acid derivatives, and nitrogen-containing heterocyclic compounds. The result showed that HarmOR30 specifically tuned to indole, rather than its analogues 3-methylindole, indole-3-carboxaldehyde, or any other structural-divergent compounds (Figure 1).

### 3.3. Functional Comparison of the HarmOR30 Orthologs

**Figure 2:**
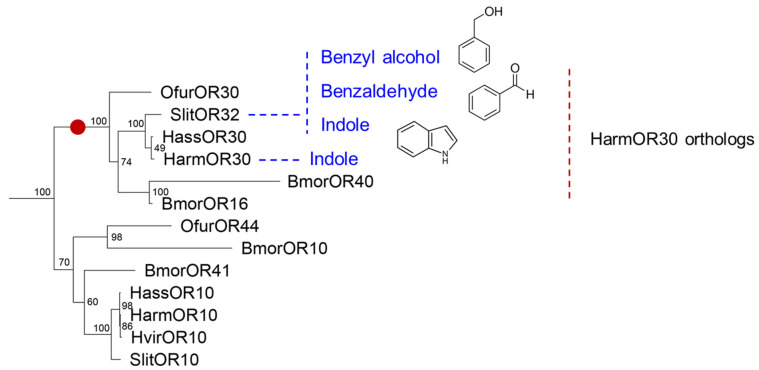
The orthologous clade of HarmOR30 from the phylogenetic tree of six lepidopteran species. The clade of HarmOR30 orthologs was marked with a red dot on the branch. Clade nodes values representing the bootstrap values were shown from 49 to 100. The ligands of functional identified ORs were shown on the right of ORs with an indigo font. *H. armigera*, Harm; *S. littoralis*, Slit; *O. furnicalis*, Ofur; *H. assulta*, Hass; *B. mori*, Bmor; *H. virescens*, Hvir.

**Figure 3:**
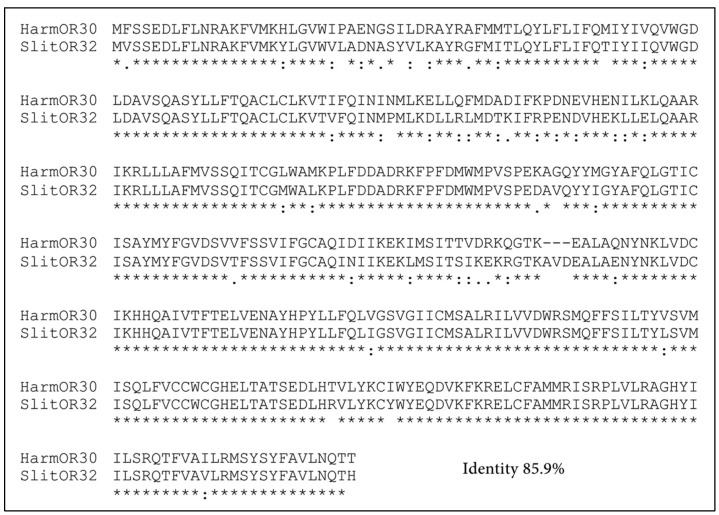
Sequences alignment of HarmOR30 and SlitOR32. Amino acid residues differences are indicated as highly (:) and moderately (.). The asterisks indicate identity across both sequences.

In Lepidoptera, functional identification of OR family has been systematically conducted in another noctuid moth *S. littoralis* by previous work, in which the tuning spectra of twenty-one SlitORs were screened against a panel of fifty-one volatiles [22]. By our previous phylogenetic studies, SlitOR32 was suggested to be the ortholog of HarmOR30 [19]. Here, we produced a coincident result by building a phylogenetic tree of 312 ORs from six lepidopteran species. The HarmOR30 orthologous clade, which contains one OR from each species of *H. armigera*, *S. littoralis*, *Ostrina furnacalis*, *H. assulta*, and two ORs from *Bombyx mori*, was identified with high node support based on bootstrap values (Figure 2). By comparing the tuning spectra of HarmOR30 and SlitOR32, we found the functional difference between the two orthologs, which share 85.9% amino acid sequence identity (Figure 3). SlitOR32 has been reported to be mainly activated by three aromatic compounds benzyl alcohol, benzaldehyde, and indole [22], whereas HarmOR30 narrowly tuned to indole and failed to respond to neither benzyl alcohol nor benzaldehyde.

## 4. Discussion

In this study, we obtained the complete coding region of three HarmORs from the proboscis of *H. armigera*, and characterized the function of HarmOR30 for sensing indole. Indole is the most widely distributed member of nitrogen-containing compounds in nature across different kingdoms and has been reported to mediate interactions among intra/inter-species of plants, microbes, invertebrates, and vertebrates [23,24,25]. In planta, indole is a derivative of shikimate pathway, resulting in biosynthesis of the essential amino acid tryptophan, a significant precursor for multifunctional and vital indole-containing secondary metabolites, such as indole-3-acetic acid (auxin), indole glucosinolates, etc. [24,26,27,28]. In addition, it can be produced as an intermediate in the biosynthesis of benzoxazinoids, a class of non-volatile defensive compounds in grasses and maize that are effective against insects and plant pathogens [24,29,30,31]. Indole can also be released as a volatile by the indole-3-glycerol phosphate lyase BX1 [32].

In maize and rice plants, indole is a specifically released herbivore-induced volatile in responding to the herbivorous attack [33,34,35]. Indole has been reported to be an essential factor in manipulating tritrophic interactions among host plants, herbivores, and its parasitoids [24,35,36]. It has been documented to be a general negative factor to the generalist herbivore *S. littoralis*, whose caterpillars and adults consistently avoid indole-producing plants for feeding or oviposition, and indole directly increases the mortality of caterpillars and reduces plant damage, despite the exposure to indole increased caterpillar growth [37]. On the other hand, certain natural enemy wasps against *S. exigua* and *S. littoralis* exploit indole to locate hosts for oviposition [35,38], whereas the story between caterpillars and wasps mediating by indole is not always reliable, as mentioned above. It was reported that tritrophic interactions can be undermined by indole, which reduces the suitability and attractiveness of caterpillars to parasitoids [38]. Within or between plants, indole was also documented as a fast and reliable priming signal to systemic tissues and neighboring plants at the early stage when there is a herbivorous attack [39,40]. It was suggested that indole is required for the priming of monoterpenes in systemic maize leaves, and enhances jasmonate-dependent defense and herbivore resistance in tea plants, likewise in other monocotyledonous and danzidicotyledonous plants [40,41,42,43].

Indole also acts as an anti-aphrodisiac to mediate the interaction between *Pieris rapae* and its parasitoid. Male *P. rapae* transfers a mixture of indole and methyl salicylate (MeS), which was generated by utilizing the amino acids tryptophan and phenylalanine as precursors, respectively, in *P. rapae*, to female at mating to curtail courtship and decrease the likelihood of female remating with conspecific males [44]. At the same time, the parasitic wasps of *P. rapae* have evolved relevant strategies by employing the anti-aphrodisiac mixture of indole and MeS to find their hosts [28,45]. It was reported that *Trichogramma* wasps were arrested after three days since *P. rapae* laid eggs on Brussels sprouts plants. Further studies showed that indole in the accessory reproductive gland secretion of mated female *P. rapae* induced changes in the foliar chemistry that arrested wasps [45]. Wasp species also exploit the anti-aphrodisiac mixture of indole and MeS of *P. rapae* by hitching a ride on a mated female butterfly, despite this behavior being innate in *T. brassicae*, whereas *T. evanescens* learns it after one successful ride on a mated female [28].

Floral scents are necessary signals mediating the interaction between plants and pollinators [46]. Indole is one of the most predominant components contributing to the floral fragrance in jasmine and tuberose [47,48] and is famous for its fragrant smell in low concentrations but the fecal smell in high concentrations. A case of indole is served as a scent signal to attract pollinators is that the wild hawkmoths *Hyles lineata* innately prefer the natural flowers of *Ipomopsis tenuituba* plants, which are light-pink and naturally emit indole and presented a stronger indole signal during the activity peak of hawkmoth [49,50]. It was demonstrated that indole attracts hawkmoths to flowers, although it has little effect on the rate at which the attracted moths probe flowers, and, combined with the high contrast (white color) of visual signals, together promote successful pollination [50].

As has been documented above, indole plays fundamental functions as a precursor, intermediate, or priming in the metabolism of organisms, as well as a crucial factor in manipulating tritrophic interactions. In *H. armigera,* HarmOR30 for sensing indole is expressed in non-olfactory organs including proboscis and legs, besides the olfactory organ antenna [18]. The particular expression pattern of HarmOR30 implies that it may play special functions in host plant evaluation. As mentioned above, maize plants specifically release indole after being attacked by herbivores such as *S. littoralis*. For *H. armigera*, maize is also hired as one of the most important host plants, the seedlings of which induce a large amount of indole when infested by *H. armigera* caterpillars [51]. HarmOR30 may be employed by the insect to detect the physiological state of plants: whether they are suffering an attack by other herbivores. On the other hand, as an essential floral scent compound in nearly thirty plant families, indole may also serve as a nectar clue for the adults of *H. armigera*, which generally bear pollen grains from a range of plant species during long-distance migration [23,52].

It is worth pointing out that HarmOR30 shows a more specific function to indole compared with its ortholog in *S. littoralis*. Despite being orthologs, their olfactory-processing function may finally lead to divergent behavior. In *S. littoralis*, no transcript of SlitOR32 was identified in other organs, including the proboscis, brain, and body except antennae [14]. On the other hand, a customized OR-expressing scheme for detecting ecological relevant cues might be employed by a specific organ in different insect species. For instance, different OR orthologs were reported to be expressed in the proboscis among three noctuid moths, respectively. In *S. littoralis*, SlitOR14 (the ortholog of HarmOR42), for sensing an aromatic floral scent phenylacetaldehyde, and SlitOR25 (the ortholog of HarmOR59), for mainly sensing acetophenone and benzyl alcohol, were identified by RT-PCR to show consistent expression patterns between both sexes [14,19,22]. However, in *M. separata*, MsepOR26 (the ortholog of HarmOR26, which mainly responds to terpene nerolidol) and MsepOR54 (the ortholog of HarmOR9) were identified by RT-PCR [15,19]. The differential expression of OR subset in proboscis may endow moths with diverse olfactory sensing functions while feeding, further promoting the differentiation of foraging preference among species.

Non-olfactory organs that are granted the odorant sensing function by specific OR have been reported by previous works. In the tobacco budworm *H. assulta*, HassOR31 is highly expressed in the female ovipositor, which thereby obtains the olfactory function for sensing (*Z*)-3-hexenyl butyrate, a volatile compound from host plants. It was suggested that gravid female moths take advantage of the odorant to find precise oviposition sites [53]. The hawkmoth *M. sexta* is a major pollinator of the wild tobacco *Nicotiana attenuata*, the flowers of which emit relatively simple floral volatile components dominated by BA [54]. The adults of *M. sexta* have coevolved a matched proboscis and exhibit an innate foraging preference to BA [3,55,56]. It was demonstrated that BA is detected only by proboscis, which is equipped with olfactory sensing neurons that express certain OR (not known yet) for detecting BA, and encourage the moth to visit a flower long enough for successful pollination [8]. The hawkmoth foraging with its slender proboscis inserts into the long corolla tube of *N. attenuate* ‘on the wing’, which costs particularly high energy [57]. Perceiving the flower scent only with the proboscis enables the moth to evaluate the specific quality of individual flowers at a very close range and significantly contributes to the moth’s net-energy gain [3,8]. However, for the cotton bollworm moth, which uptakes nectar or liquid by landing on the surface of food sources, it seems to be unnecessary to evolve a dedicated olfactory line on the proboscis. Instead, they might utilize the olfactory function of proboscis in an auxiliary form combined with the antennae.

The physiological function and behavioral effect of indole on *H. armigera* have not been reported yet. Further behavioral assays combined with functional studies in vivo of HarmOR30 should be performed in our subsequent studies, aiming to investigate the efforts of indole on development, as well as the roles of indole in host–plant recognization and assessment. In addition, it is worth getting insights into these questions: (1) What is the significance of an insect expressing the same olfactory receptor in multiple organs? (2) Does the olfactory signal of a specific odor from different organs contribute to the same physiological behavior? and (3) How does the olfactory signal from different sources get processed and integrated? Addressing these issues will contribute to our understanding of how an insect species interacts with its surrounding environment by employing specific olfactory strategies.

## Figures and Tables

**Table 1:** Primers for full-length cloning and expression vector construction.

Gene Name		Primers for Full-Length Cloning (5′-3′)
*HarmOrco*	Forward	ATGATGACCAAGGTGAAGGCCCAGG
	Reverse	TTATTTGAGTTGTACCAACACCATG
*HarmOR24*	Forward	ATGGATTCCAAAATGTCGCTGTC
	Reverse	CTACTTTGTCTGCCGAAGAACC
*HarmOR30*	Forward	ATGTTTTCTTCGGAAGATTTGT
	Reverse	TTATGTCGTCTGATTCAACACTGC
*HarmOR58*	Forward	ATGGACGTCCCTTCGTTGAAAGA
	Reverse	TTAATACATAACTGCAAAGAAAGAGT
		Primers for constructing expression vector * (5′-3′)
*HarmOR24*	Forward	ATCACTAGTGGGCCCgccaccATGGATTCCAAAATGTCGCTGTC
	Reverse	CTAGTCAGTCGCGGCCGCCTACTTTGTCTGCCGAAGAACC
*HarmOR30*	Forward	ATCACTAGTGGGCCCgccaccATGTTTTCTTCGGAAGATTTGTTTC
	Reverse	CTAGTCAGTCGCGGCCGCTTATGTCGTCTGATTCAACACTGCG

* The homologous sequences with pT7Ts Vector are underlined. Kozak sequences are shown as lowercase.

## Data Availability

All the data and resources generated for this study are included in the article and the Supplemental Materials.

## Supplementary Materials

The following supporting information can be downloaded at: https://www.mdpi.com/article/10.3390/insects13040385/s1, Table S1: Odorant compounds for functional identification of HarmORs. Table S2: Full-length sequences of HarmOR24 and OR30 cloned from the proboscis.
