# Supplementary material for: An Odorant Receptor from the Proboscis of the Cotton Bollworm Helicoverpa armigera (Lepidoptera: Noctuidae) Narrowly Tuned to Indole
Source: Insects. 2022 Apr 13;13(4):385. doi: 10.3390/insects13040385 (PMC9033110; doi:10.3390/insects13040385)
Supplement: Supplementary file 1 [file insects-13-00385-s001.zip › Supplementary Table S1-revised.pdf]

Table S1. Synthetic compounds for functional identification of HarmORs

Compound	CAS	Provider
Ethylamine	75-04-7	Sigma-Aldrich
Butylamine	109-73-9	Sigma-Aldrich
Heptylamine	111-68-2	Sigma-Aldrich
Spermidine	124-20-9	Sigma-Aldrich
Pentylamine	110-58-7	Sigma-Aldrich
1,4-Diethylbenzene	105-05-5	Sigma-Aldrich
N-methylpiperidine	626-67-5	Sigma-Aldrich
Propionaldehyde	123-38-6	Sigma-Aldrich
Butyraldehyde	123-72-8	Sigma-Aldrich
Acetic acid	64-19-7	Sigma-Aldrich
Propionic acid	79-09-4	Sigma-Aldrich
DL-lactic acid	50-21-5	Sigma-Aldrich
2,6-Di-tert-butylphenol	128-39-2	Sigma-Aldrich
2-Phenylethanol	60-12-8	Sigma-Aldrich
(*Z*)-3-hexen-1-ol	928-96-1	Sigma-Aldrich
β-Citronellol	106-22-9	Sigma-Aldrich
Geraniol	106-24-1	Sigma-Aldrich
(*Z*)-2-hexen-1-ol	928-94-9	Sigma-Aldrich
1-Heptanol	111-70-6	Sigma-Aldrich
1-Hexanol	111-27-3	Sigma-Aldrich
(*E*)-3-Hexen-1ol	928-97-2	Sigma-Aldrich
3,7-Dimethyl-3-octanol	78-69-3	Sigma-Aldrich
(1*R*)-(-)-Myrtenol	19894-97-4	Sigma-Aldrich
(-)-trans-Pinocarveol	547-61-5	Sigma-Aldrich
(-)-Linalool	126-91-0	Sigma-Aldrich
Linalool	78-70-6	Sigma-Aldrich
Benzyl alchol	100-51-6	Sigma-Aldrich
1-Octen-3-ol	3391-86-4	Sigma-Aldrich
(*E*)-2-Hexen-1-al	6728-26-3	Sigma-Aldrich
(1*R*)-(-)-Myrtenal	18486-69-6	Sigma-Aldrich
Heptanal	111-71-7	Sigma-Aldrich
Phenylacetaldehyde	122-78-1	Sigma-Aldrich
2-Heptanone	110-43-0	Sigma-Aldrich
2,3-Butanedione	431-03-8	Sigma-Aldrich
(*R*)-(+)-Limonene	5989-27-5	Sigma-Aldrich
α-Pinene	80-56-8	Sigma-Aldrich
α-Humulene	6753-98-6	Sigma-Aldrich
(-)-trans-Caryophyllene	87-44-5	Sigma-Aldrich
Cumene	98-82-8	Sigma-Aldrich
Sabinene	3387-41-5	Sigma-Aldrich
Methyl Salicylate	119-36-8	Sigma-Aldrich
Ethyl acetate	141-78-6	Sigma-Aldrich
Methyl jasmonate	39924-52-2	Sigma-Aldrich
Butyl salicylate	2052-14-4	Sigma-Aldrich
Nonanal	124-19-6	Sigma-Aldrich
Methyl eugenol	93-15-2	Sigma-Aldrich
(1*S*)-(-)-Verbenone	1196-01-6	Sigma-Aldrich
(*S*)-cis-Verbenol	18881-04-4	Sigma-Aldrich
Methyl benzoate	93-58-3	Sigma-Aldrich

Myrcene	123-35-3	Sigma-Aldrich
(-)-β-Pinene	18172-67-3	Sigma-Aldrich
(S)-(-)-Limonene	5989-54-8	Sigma-Aldrich
α-Terpinene	99-86-5	Sigma-Aldrich
(-)-Caryophyllene oxide	1139-30-6	Sigma-Aldrich
Farnesene, mixture of isomers	502-61-4	Sigma-Aldrich
4-Ethylbenzaldehyde	4748-78-1	Sigma-Aldrich
3-Vinylbenzaldehyde	19955-99-8	Sigma-Aldrich
Benzaldehyde	100-52-7	Sigma-Aldrich
(*Z*)-3-Hexenyl acetate	3681-71-8	Sigma-Aldrich
(*E*)-2-Hexenyl acetate	2497-18-9	Sigma-Aldrich
4'-Ethylacetophenone	937-30-4	Sigma-Aldrich
Cinnamaldehyde	104-55-2	Sigma-Aldrich
(±)-Citronellal	106-23-0	Sigma-Aldrich
Benzyl acetate	140-11-4	Sigma-Aldrich
2-Pentadecanone	2345-28-0	Sigma-Aldrich
Cedrol	77-53-2	Sigma-Aldrich
Indole	120-72-9	Sigma-Aldrich
3-Methylindole	83-34-1	Sigma-Aldrich
Indole-3-carboxaldehyde	487-89-8	Sigma-Aldrich
Benzyl acetone	2550-26-7	Sigma-Aldrich
Salicylaldehyde	90-02-8	Sigma-Aldrich
4-Methoxybenzyl alcohol	105-13-5	Sigma-Aldrich
4-Hydroxy-4-methyl-2-pentanone	123-42-2	Sigma-Aldrich
Methyl phenylacetate	101-41-7	Sigma-Aldrich
Eugenol	97-53-0	Sigma-Aldrich
1-Octanol	111-87-5	Sigma-Aldrich
Ocimene	13877-91-3	Sigma-Aldrich
cis-Jasmone	488-10-8	Sigma-Aldrich
δ-Decanolactone	705-86-2	Sigma-Aldrich
Geranyl acetate	105-87-3	Sigma-Aldrich
