# Supplementary material for: An Odorant Receptor from the Proboscis of the Cotton Bollworm Helicoverpa armigera (Lepidoptera: Noctuidae) Narrowly Tuned to Indole
Source: Insects. 2022 Apr 13;13(4):385. doi: 10.3390/insects13040385 (PMC9033110; doi:10.3390/insects13040385)
Supplement: Supplementary file 1 [file insects-13-00385-s001.zip › Supplementary Table S2-revised.pdf]

Table S2: Full-length sequences of HarmOR24 and OR30 cloned from the proboscis

Gene Name	Identity with Reference Gene	Transmembrane domains	Amino Acid Sequences
HarmOR24	99.5%	7	MDSKMSLSSASLATHLRLLRWCGYCRLAGGARLSRLHALYRALTLALTTVYLLQ<br>ECVYAYQVQQDMDKLARVMFLLLCHITSIAKQLVFHLKAERIDEMLAGLEDPLY<br>NQPEEAHRRLLGATAASASRFVRAYSGCAVVTCTLWITFPVMYRLQGLPVEFPF<br>WITVDYNRPTMFILVLAYSYYVTTLVGIANTTMDAFMATVLNQCKTQLRLLRMN<br>FECLPERAAALSRQLGGSYDAALFALFRECLVHYEKITETAKMLQNIFGTAILIQF<br>GIGGWILCMAAYKIVSLNMLSVEFASMALFISCILTELFLYCYYGNEVTDESERVS<br>QSLYSMEWRRARLTFRRSLVLVMERAKRPLRPAAGRVIPLSLDTFVKIIKSSYTFYA<br>VLRQTK-
HarmOR30	97.4%	7	MFSSEDLFLNRAKFVMKHLGVWIPAENGSILDRAYRAFMMTLQYLFLIFQMIYIV<br>QVWGDLDAVSQASYLLFTQACLCLKVTIFQININMLKELLQFMDADIFKPDNEV<br>HENILKLQAARIKRLLLAFMVSSQITCGLWAMKPLFDDADRKFPFDMWMPVSPE<br>KAGQYYMGYAFQLGTICISAYMYFGVDSVVFSSVIFGCAQIDIIKEKIMSITTVDRK<br>QGTKEALAQNYNKLVDCIKHHQAIVTFTELVENAYHPYLLFQLVGSVGIICMSA<br>LRILVVDWRSMQFFSILTYVSVMISQLFVCCWCGHELTATSEDLHTVLYKCIWYE<br>QDVKFKRELCFAMMRISRPLVLRAGHYIILSRQTFVAILRMSYSYFAVLNQTT-
